# Toward early estimation and treatment of addiction vulnerability: radial arm maze and N-acetyl cysteine before cocaine sensitization or nicotine self-administration in neonatal ventral hippocampal lesion rats

**DOI:** 10.1007/s00213-016-4421-8

**Published:** 2016-09-17

**Authors:** Kalyan N. Rao, Alena M. Sentir, Eric A. Engleman, Richard L. Bell, Leslie A. Hulvershorn, Alan Breier, R. Andrew Chambers

**Affiliations:** 1 Department of Psychiatry, Indiana University School of Medicine, Indianapolis, IN USA; 2 IU Neuroscience Research Center, Indianapolis, IN USA; 3 Adolescent Dual Diagnosis Clinic, Riley Hospital for Children at IU Health, Indianapolis, IN USA; 4 Department of Psychiatry, Psychotic Disorders Program at IU, Indiana University School of Medicine, Indiana University Prevention and Recovery Center for Early Psychosis (PARC), Indianapolis, IN USA; 5 Laboratory for Translational Neuroscience of Dual Diagnosis & Development, Indiana University School of Medicine, Suite 314, 320 West 15th Street, Indianapolis, IN 46202 USA; 6 Addiction Psychiatry Training Program, IU Neuroscience Research Center, Indiana University School of Medicine, Suite 314, 320 West 15th Street, Indianapolis, IN 46202 USA

**Keywords:** Schizophrenia, Neurodevelopment, Dual diagnosis, Radial arm maze, Cocaine, Behavioral sensitization, N-acetyl cysteine, Adolescence

## Abstract

**Rational:**

Prefrontal cortical (PFC)–hippocampal–striatal circuits, interconnected via glutamatergic signaling, are dysfunctional in mental illnesses that involve addiction vulnerability.

**Objectives:**

In healthy and neurodevelopmentally altered rats, we examined how Radial Arm Maze (RAM) performance estimates addiction vulnerability, and how starting a glutamatergic modulating agent, N-acetyl cysteine (NAC) in adolescence alters adult mental illness and/or addiction phenotypes.

**Methods:**

Rats with neonatal ventral hippocampal lesions (NVHL) vs. SHAM-operated controls were randomized to NAC vs. saline in adolescence followed by cognitive testing (RAM) in early adulthood and then cocaine behavioral sensitization (experiment 1; *n* = 80) or nicotine self-administration (experiment 2; *n* = 12).

**Results:**

In experiment 1, NVHL rats showed over-consumption of food (Froot-Loops (FL)) baiting the RAM with poor working memory (low-arm entries to repeat (ETR)), producing an elevated FL to ETR ratio (“FLETR”; *p* < 0.001). FLETR was the best linear estimator (compared to FL or ETR) of magnitude of long-term cocaine sensitization (*R* ^2^ = 0.14, *p* < 0.001). NAC treatment did not alter FL, ETR, FLETR, or cocaine sensitization. In experiment 2, FLETR also significantly and uniquely correlated with subsequent drug seeking during nicotine-induced reinstatement after extinction of nicotine self-administration (*R* ^2^ = 0.47, *p* < 0.01). NAC did not alter RAM performance, but significantly reversed NVHL-induced increases in nicotine seeking during extinction and reinstatement.

**Conclusions:**

These findings demonstrate the utility of animal models of mental illness with addiction vulnerability for developing novel diagnostic measures of PFC–hippocampal–striatal circuit dysfunction that may reflect addiction risk. Such tests may direct pharmacological treatments prior to adulthood and addictive drug exposure, to prevent or treat adult addictions.

## Introduction

Substance use disorders are concentrated in mentally ill people (Regier et al. 1990) producing greater psychiatric and medical morbidity, risk of incarceration, homelessness, and early death (Drake et al. 2001; Owen et al. 1996). Preclinical models show that dysfunctional cortical–striatal–temporal–limbic circuitry like that in mental illness enhances drug-reinforcement and addiction vulnerability (Chambers et al. 2001, 2010a). Specifically, early ventral hippocampus damage in rats, resulting in abnormal connectivity and functionality of the prefrontal cortex (PFC) and nucleus accumbens, generates adult psychiatric symptoms while lowering thresholds for acquiring addiction (Chambers 2007; Chambers et al. 2007; Chambers and Self 2002). Meanwhile, longitudinal human studies show that heritable, childhood “externalizing” phenotypes lead to both adult mental illness and substance disorders (Iacono et al. 2008; Malone et al. 2002; Tarter et al. 2003; Zucker 2008).

Glutamate neurotransmission mediates communication between the ventral hippocampus, PFC, and nucleus accumbens (Kelley and Domesick 1982; O’Donnell et al. 1999; Pennartz et al. 1994). Dopamine-facilitated plasticity at glutamate synapses in the nucleus accumbens invokes healthy and pathological changes in motivated behavior (Chambers et al. 2007; Goto and Grace 2005; Grace et al. 2007; Kauer and Malenka 2007). Accordingly, dysfunction of cortical–striatal circuits that utilize glutamate may underpin mental illness (Adler et al. 1999; Goff and Coyle 2001; Jentsch and Roth 1999), addiction (Kalivas 2009; Rao et al. 2015), and their linkage (Chambers et al. 2001, 2007). In testing this theory, we examined radial arm maze (RAM) measures of PFC–hippocampal-dependent cognition (Floresco et al. 1997), and the effects of N-acetyl cysteine (NAC), a glutamate-modulating agent (Baker et al. 2002) on addiction-related behaviors in neonatal ventral hippocampal lesion (NVHL) rats.

The NVHL model of schizophrenia (Lipska et al. 1993) is produced by delivering ibotenic acid to the ventral hippocampus of 7-day old rats, approximating the third trimester of human development (Clancy et al. 2007). NVHL rats show peri-adolescent onset of positive-like symptoms associated with schizophrenia and “externalizing” disorders, including behavioral hyperactivity to stress and novelty that is treatable with neuroleptics (Lipska and Weinberger 1994). They also show prodromal social deficits (Sams-Dodd et al. 1997) and cognitive impairment reflecting PFC–hippocampal network dysfunction that is refractory to neuroleptics (Bahner et al. 2015; Brady et al. 2010; Chambers et al. 1996; Tek et al. 2002). Molecular, cellular, and functional abnormalities related to PFC glutamate neurotransmission in NVHL rats parallel neuroimaging and histopathological findings in schizophrenia (Chambers et al. 2013; Tseng et al. 2009). Replicating extreme rates of addiction in schizophrenia (Barnett et al. 2007; Volkow 2009), NVHL rats are abnormally sensitive to the activating and reinforcing effects of cocaine, nicotine, and alcohol (Berg and Chambers 2008; Berg et al. 2011, 2014; Chambers and Self 2002; Chambers and Taylor 2004; Conroy et al. 2007).

Using NVHL and SHAM-operated rats, we conducted two developmental–longitudinal experiments that started N-acetyl-cysteine (NAC) treatment in adolescence, and measured adult RAM-cognitive performance profiles before behavioral sensitization to cocaine (experiment 1), or nicotine self-administration (experiment 2). NAC is a pro-drug for glutathione, which protects neurons from cumulative oxidative stress, and cysteine, which, via glial cysteine/glutamate transporters, raises extra-synaptic levels of glutamate (Asevedo et al. 2014; Cabungcal et al. 2014; Lavoie et al. 2008; Moran et al. 2005). This mechanism increases glutamatergic tone onto cortical-striatal axonal terminals that bear metabotropic glutamate receptors (mGluR2/3), which dampens stimulus-induced glutamate release, e.g., corresponding to drug-related cues, and craving, or glutamate dysregulation in mental illness (Carmeli et al. 2012; Knackstedt et al. 2010; Kupchik et al. 2012; Moussawi et al. 2009). Interposing RAM-testing between adolescent NAC treatment initiation and adult addiction-related behavioral phenotyping allowed testing of potential effects of NAC on RAM performance, and exploration of how RAM performance could estimate severity of subsequent addiction-related behavior.

## Materials and methods

### Subjects, neonatal surgery, drugs, and experimental design

Pregnant Sprague-Dawley rats (Harlan, Indianapolis) arriving at 14- to 16-days gestation, were housed on a 12-h light cycle. Male pups (15–18 g; PD 7) received ibotenate (3 μg) or vehicle (0.3 μl) into the ventral hippocampus bilaterally (AP −3.0, ML ±3.5, VD −5.0 mm/bregma), detailed in (Chambers and Lipska 2011). Pups were returned to litters balanced closely to 5:4 ratios of lesioned to SHAMs. After weaning (PD 21), pups were pair-housed (same lesion) then singly housed at the beginning of RAM (PD 55–63). Different rats were prepared for experiments 1 and 2. Animal care complied with the *Guide for the Care and Use of Laboratory Animals,* and Indiana University IACUC.

**Fig. 1:**
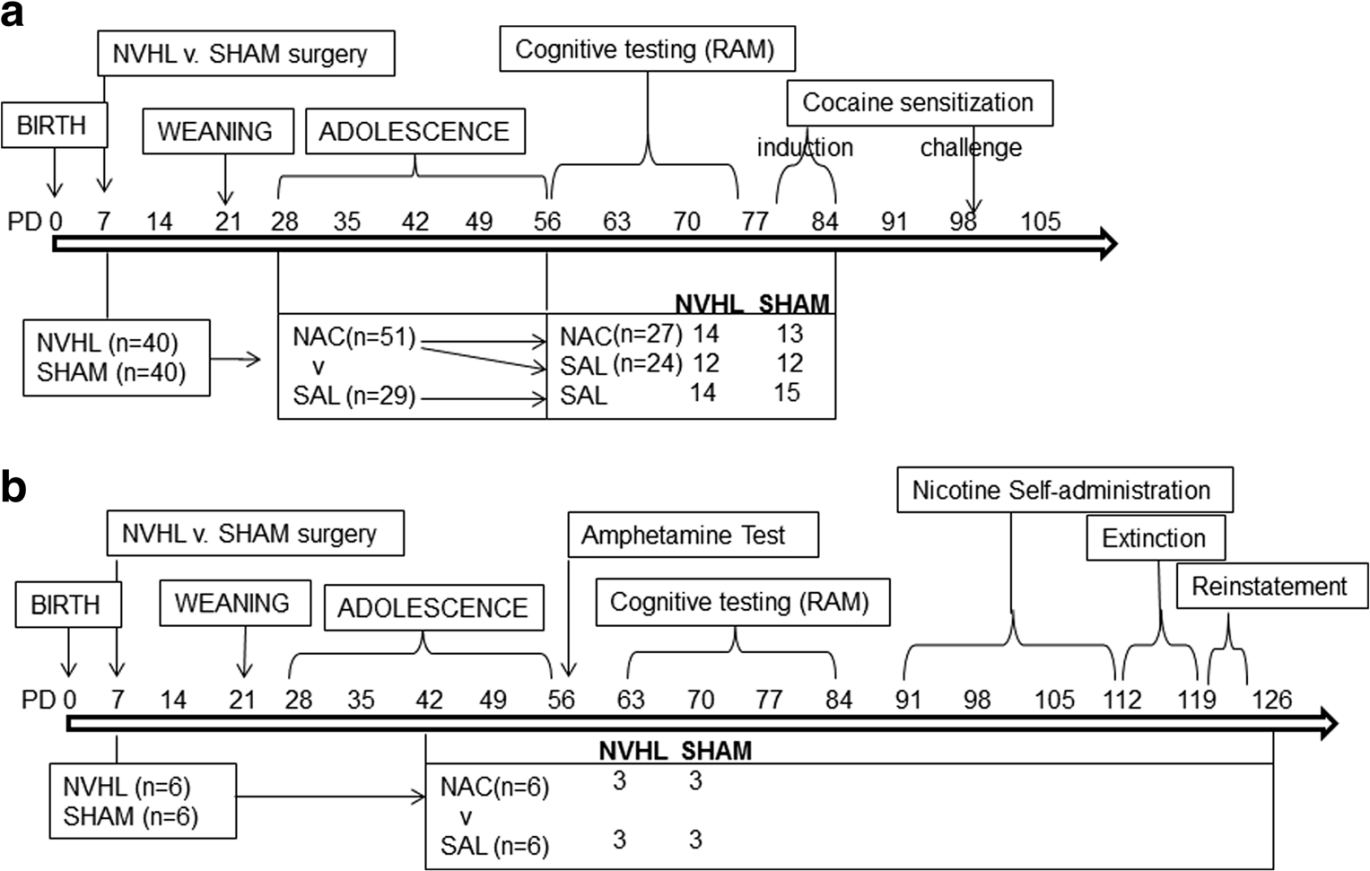
Experimental schedule for experiments 1 (**a**) and 2 (**b**). Postnatal Day (PD) timing of experimental interventions and N-acetyl cysteine (NAC) dosing according to treatment groups and numbers of animals per subgroup (*n*) are shown

NAC (Sigma, St. Louis, MO, USA) was prepared in saline, NaOH adjusted to pH 7.2 at 100 mg/ml (shown to modulate cocaine-seeking in rats (Reichel et al. 2011)). Rats were randomized to daily NAC (100 mg/kg ip) or saline (SAL; 1 ml/kg) injections, 60–90 min before behavioral testing. For experiment 1 (Fig. 1a), Cocaine (NIDA) was prepared in stock (15 mg/ml) and injected at 15 mg/kg ip. For experiment 2 (Fig. 1b), d-amphetamine sulfate (Sigma) was injected at 1.5 mg/kg ip (Lipska et al. 1993). Nicotine hydrogen tartrate salt (Sigma) was prepared in 0.9 % sterile saline to make a 0.5 mg/ml stock (base of the salt, pH adjusted to 7.4 (Matta et al. 2007)). Self-administration concentrations were prepared per rat weight from stock as were the 0.5 or 0.25 mg/kg of nicotine for reinstatement. In experiment 1, rats began daily injections on PD 28 (early adolescence; Bell et al. 2014). About twice as many rats entered NAC compared to SAL treatment so that the NAC group could be further randomized (PD 56) to saline (NAC-SAL), or NAC continuation ((NAC-NAC) through RAM (PD 56–74) until the end of initial cocaine sensitization (PD 81–85). In experiment 2 (Fig. 1b), NAC or SAL began in mid-adolescence (PD 42), and continued through locomotor testing, RAM, and nicotine self-administration (PD 91–122).

### Radial arm maze (experiments 1 and 2)

One day before and during RAM, rats were food restricted to support reward seeking. Rat chow (Harlan; 1–2 bricks (5 g)) was delivered to home cages after sessions to maintain body weights at 15 % from pre-RAM weights. The RAM, described in detail in (Berg et al. 2015), had eight 10 × 60-cm runways extending radially from a central arena (50-cm diameter). For each session, a receptacle at the end of all 8 arms was pre-loaded with 1/2 of a Kellogg’s Froot Loop®. After starting from the central arena, rats explored the maze until either all arms were entered (four paws in) or 300-s elapsed. The investigator, blind to group, recorded three dependent measures: (1) entries to repeat (ETR), number of arms entered without a repeat (measure of working memory); (2) session time, total time to enter all arms (measure of performance efficiency); and (3) total number of Froot Loops (FL) consumed (measure of appetitive motivation). FL ranged from 0 to 8, never exceeded arms entered, but could be greater or less than ETR.

### Cocaine sensitization (experiment 1) and locomotor activation (experiment 2)

Locomotor recording was measured in 43.2 × 43.2 × 30.5-cm arenas equipped with infrared beams (Med Associates, described in (Chambers and Taylor 2004). Initial cocaine sensitization was assessed in 2-h, daily sessions for 5 days, with cocaine delivered after 1 h, and a challenge session 2 weeks later. Experiment 2 rats were tested in a single 3-h session for measuring locomotion to the novel environment (hour 1), a saline injection (1 ml/kg ip @ 60 min) and amphetamine (1.5 mg/kg ip @120 min).

### Nicotine self-administration (experiment 2)

After a week of recovery from jugular catheter implantation (see (Berg et al. 2014)) rats were food restricted for 15 sessions of acquisition of nicotine self-administration (two-lever Med Associates chambers detailed previously (Berg et al. 2014)). Two-hour sessions started with house lights on to signal drug availability with a single priming infusion of nicotine. Responses on the nicotine-paired lever turned house lights off and a cue light above the nicotine lever on for the 3-s infusion (0.015 mg/kg nicotine in 0.050 ml saline (FR1)). A 17-s time out began after infusion when presses had no effects and all lights were out. After acquisition and 2 days off, rats entered 5 days of 2-h extinction sessions when catheters were not connected, and lever presses produced no infusions. After extinction and 2-days off, rats underwent 3-days of 2-h reinstatement sessions (catheters not connected) in which rats were given injections, 30 min before, of saline (day 1, 1 mg/kg ip), and nicotine (day 2, 0.25 mg/kg ip; day 3, 0.5 mg /kg).

### Data analysis and lesion verification

**Fig. 2:**
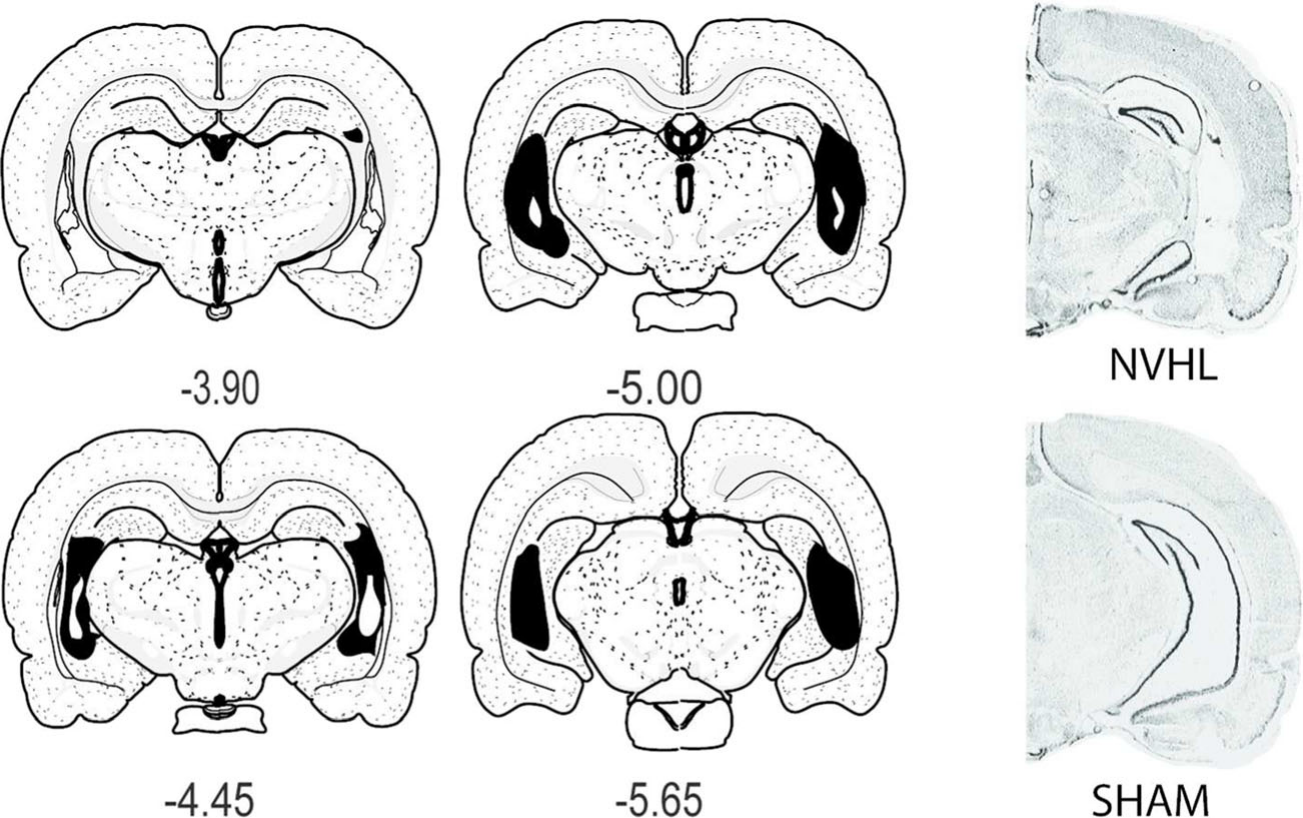
Representative maps and photomicrographs of rats included in the study. *Left four panels* show greatest (*solid black*) and least (*white within the black area*) extent of tissue damage in NVHL rats included in the study in coronal sections (in millimeter relative to Bregma; Swanson 2004). The *micrographs at right* shows an example of histology of a NVHL compared to a SHAM brain where lateral ventricular enlargement and atrophy of primary ventral hippocampal cell layers are observable at low magnification

For experiment 1, initial analyses utilized omnibus two-way repeated measure ANOVAs (independent factors of lesion (NVHL vs. SHAM) and drug (three levels: SAL, NAC-SAL, NAC-NAC)). For RAM, dependent measures (ETR, session time, FL) were across bins (means of three consecutive daily sessions). For cocaine sensitization, day 1 and challenge data were examined as a repeated measure of pre- vs. post-injection locomotion, while sensitization over the 5-days examined post-injection activity. Post hoc testing examined interactions between main effects or main effects and time, with unitary ANOVAs and Bonferroni corrections as appropriate. In experiment 2, similar two-way, repeated measure ANOVAs examined lesion and drug (SAL vs. NAC). In the locomotor session, the hour was the repeated measure whereas in RAM and nicotine self-administration, bins and days were repeated. All significant (*p* < 0.05) results (and important *p* > 0.05, NS, findings) are reported with data presented as means ± SEM. Linear regressions examined relationships between RAM and cocaine sensitization or nicotine self-administration. Only rats with brains revealing accurate lesions by pre-established criteria, assessed blind to behavioral results (detailed in (Chambers and Lipska 2011)) were analyzed. In experiment 1, 8 of 48 brains, and in experiment 2, 2 of 8 had disqualifying lesions and were excluded. Figure 2 shows mapping of lesions among NVHL rats (*n* = 40 in experiment 1; *n* = 6 in experiment 2) included in the study.

## Results

### Experiment 1: RAM and cocaine behavioral sensitization

**Fig. 3:**
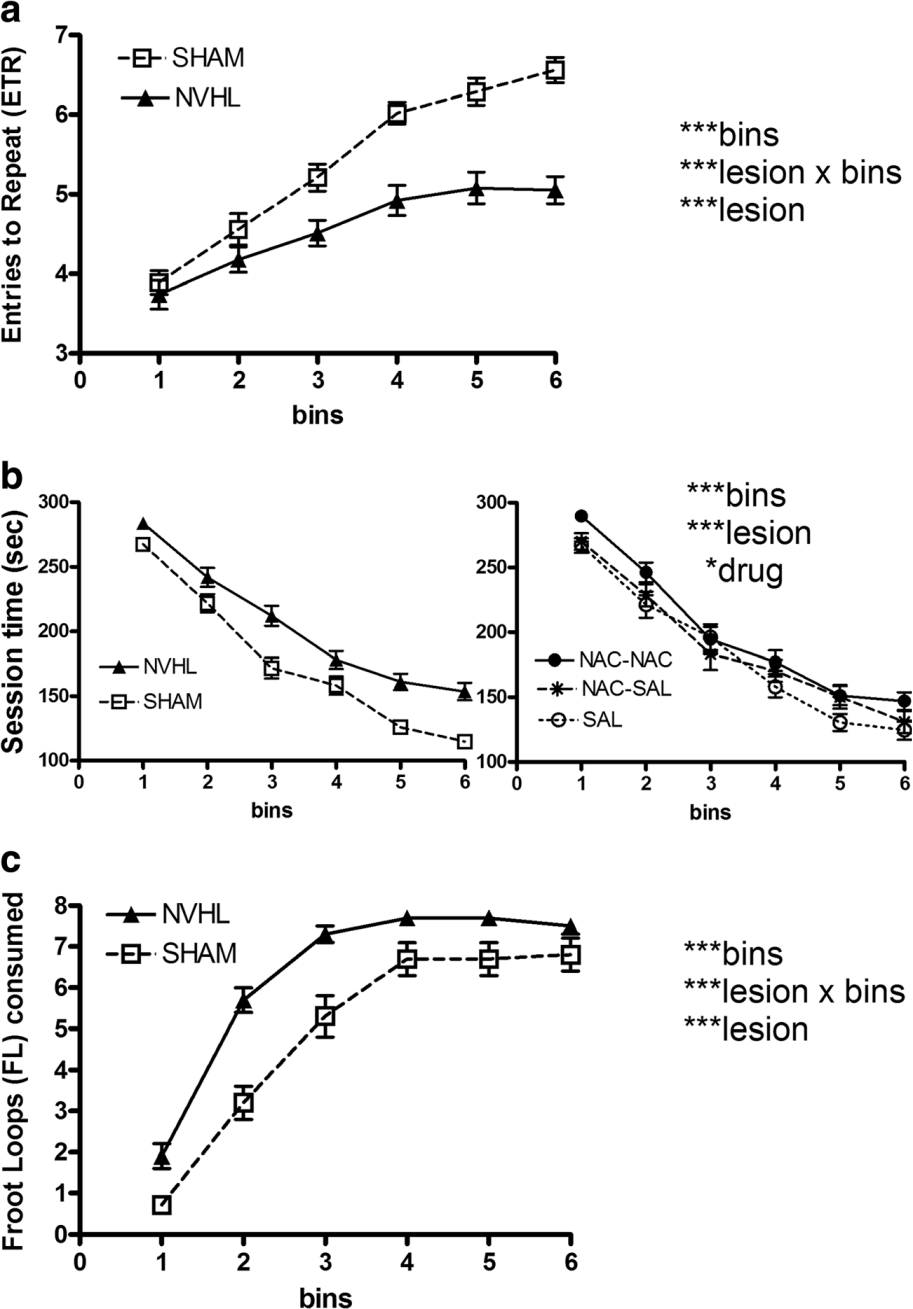
Cognitive testing on the radial arm maze (RAM; experiment 1). For each dependent measure: (**a** entries to Repeat; **b** session time; **c** Froot Loops consumed)), drug treatment and lesion status were analyzed as independent factors in the same mixed ANOVA, across the six bins (18 days; 3 days per bin) of testing. Data are shown primarily according to lesion grouping only, given that significant lesion-based differences were identified across all three dependent performance measures. In the *left panel* of **b,** the data is grouped by lesion status, whereas on the *right*, the same data is grouped according to drug group only to allow visualization of the main effect of drug treatment. Total rats and subgroup numbers examined are shown in Fig.1. Data are depicted as means ± SEMs. All significant main effects and interactions of the mixed ANOVA for each dependent variable are shown at *right* with significance levels denoted as *p < 0.05, ** *p* < 0.01, and *** *p* < 0.001

One day prior to RAM (∼PD 56), NVHL rats (*n* = 40) weighed 278.7 ± 2.8 g, compared to SHAMS (*n* = 40; 286.9 ± 2.9 g). This 3 % difference was significant (lesion: *F*(1,74) = 4.3, *p* < 0.05), while NAC had no effect, or interaction with NVHLs on weight. Rats showed learning on the RAM with increasing ETR over sessions (bins: *F*(5, 370) = 49.0, *p* < 0.001; Fig. 3a). However, NVHLs were impaired with lower ETR (lesion: *F*(1, 74) = 44.4, *p* < 0.001) and deficient ETR across sessions (lesion × bins: *F*(5, 370) =5.4, *p* < 0.001). NAC treatment had no effect or interactions in altering ETR. For session time (Fig. 3b), overall improvement in maze performance occurred (bins: *F*(5370) = 205, *p* < 0.001), but NVHLs were slower (NVHL vs. SHAM comparison: Fig. 3b, left; lesion: *F*(1,74) = 25.5, *p* < 0.001). However, NVHLs did show improvement at rates that were not different from SHAMs (lesion × bins: *F*(5,370) = 2.1, NS). NAC treatment did show a significant main effect in altering session time (drug: *F*(2,74) = 3.4, *p* < 0.05) without interactions. NAC dosing group comparisons are shown in Fig. 3b, right. Rats treated with saline tended to be more efficient in completing the maze (mean session time/bin, 182.9 + 5.5 s) compared to their NAC-SAL (188.9 + 6.5 s) and NAC-NAC (201.0 + 4.7 s) counterparts, although post-hoc analyses (one-way ANOVA/Bonferroni) did not tease out a significant difference between any two regimens. Across bins, there were overall increases in FL eating (bins: *F*(5370) = 259.4, *p* < 0.001; Fig. 3c). Notably, despite NVHL deficits in cognitive performance, NVHL rats showed greater FL consumption (lesion: *F*(1,74) = 15.4, *p* < 0.001), particularly in the earlier bins (lesion × bins: *F*(5370) = 5.2, *p* < 0.001). NAC did not impact FL consumption.

**Fig. 4:**
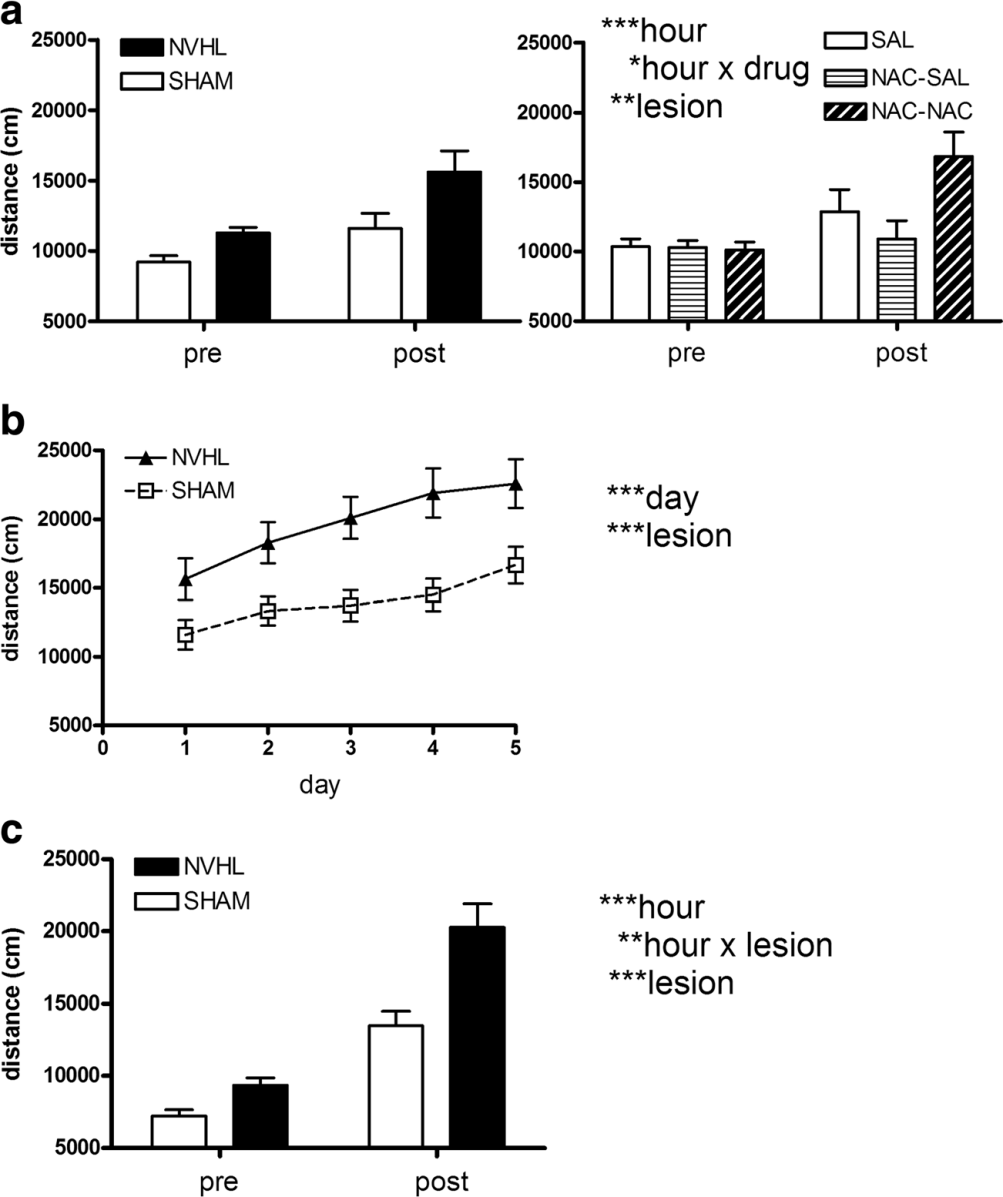
Cocaine sensitization (experiment 1). Data are shown primarily according to lesion grouping only, given that significant lesion-based differences were identified across all three stages of behavioral sensitization. Pre and post-injection locomotor distance (per hour) surrounding the first cocaine injection (**a**) is shown according to lesion grouping in the *left panel*, with the same data grouped according to drug treatment in the *right panel*. Short-term sensitization over the first 5 days of cocaine injections (growth of post-injection distance) (**b**) and pre and post-injection locomotor distance in the cocaine challenge session (**c**) (to detect long-term sensitization) reveal robustly significant lesion effects. Total rats and subgroup numbers examined are same shown in Fig. 1. Data are depicted as means ± SEMs. All significant main effects and interactions of the mixed ANOVA are shown at right with significance levels denoted as *p < 0.05, ** *p* < 0.01, and *** *p* < 0.001

In behavioral sensitization, cocaine increased locomotion on day 1 (Fig. 4a; hour: *F*(1,74) = 14, *p* < 0.001). There was an effect of NVHL to increase locomotion generally (lesion: *F*(1,74) = 8.2, *p* < 0.01), but without a lesion × hour interaction. Figure 4a, left, shows lesion group comparisons; Fig. 4a, right, shows NAC group comparisons. NAC did impact cocaine’s stimulating locomotor response (drug × hour: *F*(2,74) = 4.1, *p* < 0.05), but NAC had no effect or interactions with NVHL. This effect was further observed in post hoc testing (one way ANOVAs of drug regimen on pre-injection and post-injection locomotion separately). There were no differences in pre-injection locomotion by NAC regimen, but post-injection, the three groups differed significantly (*F*(2,77) = 3.5, *p* < 0.05), with NAC-SAL and NAC-NAC groups showing the lowest vs. highest post-injection locomotion respectively (Bonferroni, *p* < 0.05). Figure 4b shows cocaine sensitization over the 5 days. There was a significant behavioral sensitization effect (day: *F*(4296) = 7.6, *p* < 0.001). Again, NVHL rats showed increased cocaine-induced activation throughout the series (lesion: *F*(1,74) = 18.8, *p* < 0.001 with lesion × day: *F*(4296) = 0.7, *p* = NS), while NAC regimen had no main effect or interactions. In the cocaine challenge session, cocaine increased locomotion (hour: *F*(1,74) = 110.9, *p* < 0.001; Fig. 4c). NVHLs showed overall increased locomotion (lesion: *F*(1,74) = 13.8, *p* < 0.001), and greater cocaine-induced increases (lesion × hour: *F*(1,74) = 7.5, *p* < 0.01). NAC history produced no main effect or interactions.

### RAM performance as an estimator of cocaine sensitization

**Fig. 5:**
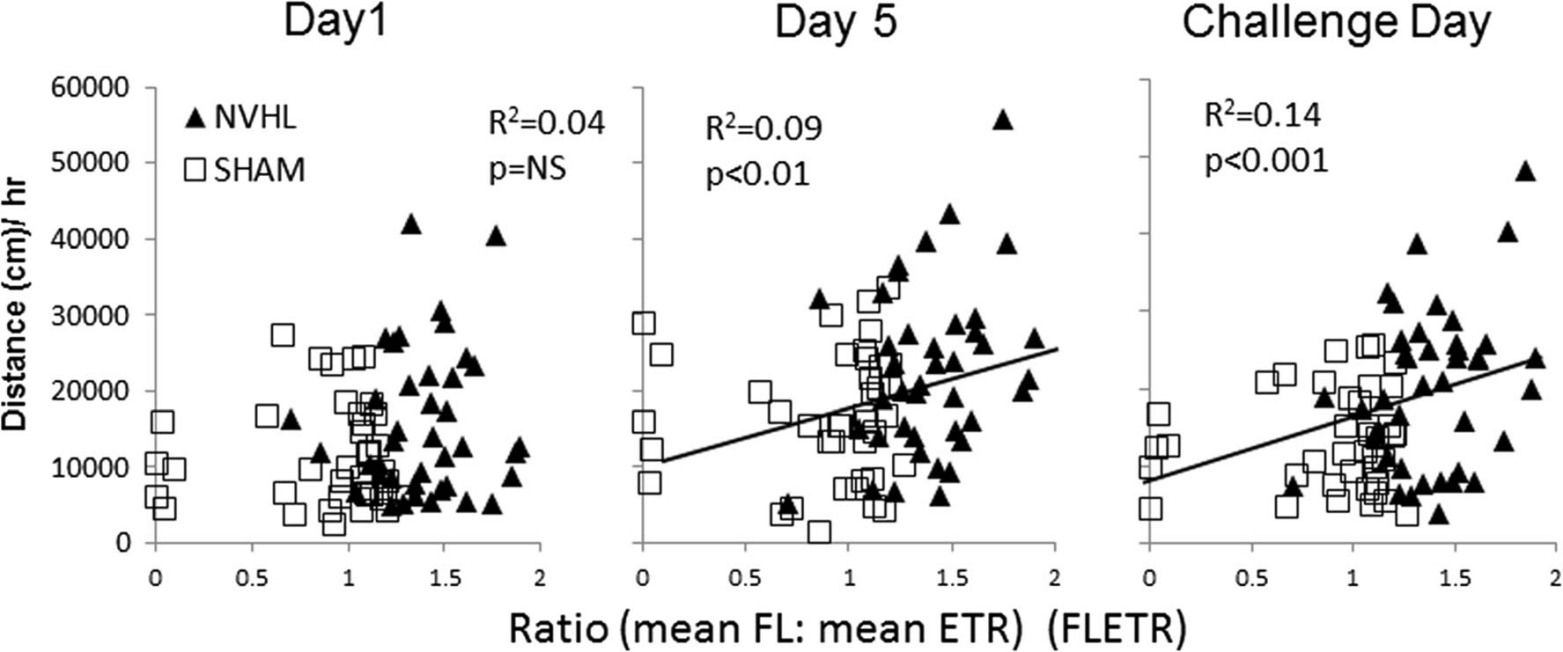
RAM performance as linear estimator of subsequent cocaine sensitization (experiment 1). Scatter plots show post-cocaine locomotor activation (*y*-axis) plotted for each rat as a function of its RAM performance calculated as the ratio of mean Froot loops consumed to mean ETR recorded across the six bins (FLETR; *x*-axis). Over the entire population (*N* = 80), *R* ^2^ values and significance levels of the linear regression (*slope*) characterizing a relationship between FLETR and subsequent cocaine-induced locomotion strengthens with more cocaine injections, rightward across panels, as the measured locomotor activation levels represent increasing contributions encoded by the behavioral sensitizing effects of cocaine. The best fit linear equations for day 5 and challenge day sessions were *y* = 7,539*x* + 10,979, and *y* = 8,334*x* + 7,310, where *y* = cocaine-induced locomotion (in centimeter) and *x* = FLETR

For all *N* = 80 rats, we calculated mean ETR, mean FL consumed, and ratio of mean FL to mean ETR (“FLETR”), over the 18 RAM sessions (Table 1, left). Mean FL and mean ETR were not linearly associated (*R* ^2^ = 0.002, *p* = NS) with each other. As already described, ETR, and FL were not altered by NAC, and we further confirmed that mean FLETR was also not altered by NAC as a main effect or interaction. In contrast, the effect of lesion on mean FLETR was significant (*F*(1,74) = 48, *p* < 0.001). Also, simple *t* tests showed that NVHLs were significantly different from SHAMs across FL, ETR and FLETR (*p* < 0.001), with the largest difference (% change between SHAMs and NVHLS) occurring with FLETR (+57.3 %), compared to +28.6 % for FL and −15.8 % for ETR; Table 1, left). Linear regressions performed between each of these RAM variables and post-injection locomotion across progressive stages of cocaine sensitization (day 1, day 5, challenge day) are shown in Table 1, right. FLETR was a better correlate (highest *R* ^2^; greater significance) of cocaine activation than either FL or ETR alone on any given day of sensitization, and the correlation of FLETR with post-cocaine locomotion improved as the number of cocaine sensitization sessions increased (Fig. 5). Also as suggested in Fig. 5 plots, variance associated with FLETR in both SHAM and NVHL groups contributed to this relationship. Neither NVHL nor SHAM subgroups taken alone produced significant linear regressions; outlier analysis testing the removal of the five SHAM animals on the leftmost range of the challenge data also did not change the strength of the linear correlation (*N* = 75: *R* ^2^ = 0.14, *p* < 0.001).

**Table 1:** RAM-dependent measures and relationship with cocaine behavioral sensitization experiment 1

Variable				RAM (FLETR) linear regression with post-cocaine injection locomotion		
	(*n* = 40)	(*n* = 40)		(*N* = 80)		
	SHAM	NVHL	*t*	Day 1	Day 5	Challenge
FL	4.89 ± 0.32	6.29 ± 0.13	6.6***	*R* ^2^ = 0.03	*R* ^2^ = 0.04	*R* ^2^ = 0.06
				NS	NS	m = 1,346*
						*b* = 9,330**
ETR	5.4 ± 0.09	4.6 ± 0.09	−4.0***	*R* ^2^ = 0.01	*R* ^2^ = 0.08	*R* ^2^ = 0.09
				NS	*m* = −4,107*	*m* = −3,874**
					*b* = 40,150***	*b* = 36,229***
FLETR	0.89 ± 0.06	1.40 ± 0.04	7.1***	*R* ^2^ = 0.04	*R* ^2^ = 0.09	*R* ^2^ = 0.14
				NS	*m* = 7,539**	*m* = 8,334***
					*b* = 10,979***	*b* = 7,310**

Significance levels of simple *t* tests and linear regression modeling (*x*, RAM variable; *y* = post-cocaine locomotion (in centimeter), *x* = FLETR, *m* = slope, *b* = *y* intercept) are reported as *p < 0.05, **p < 0.01, and ***p < 0.001 with *NS* denoting non-significance. *R* ^2^ values reporting goodness of fit, are regarded a significant as per the slope

### Experiment 2: locomotor stimulation, RAM and nicotine self-administration

Rats (*n* = 12) first tested in a 3-h locomotor activation test showed significant overall differences in locomotion by time (hours: *F*(2, 16) = 12.2, *p* = 0.001) reflecting different responses to novelty (hour 1, 10,364 ± 873 cm), saline (hour 2, 5,105 ± 473 cm), and amphetamine(hour 3, 10,094 ± 1,238 cm). However, there were no significant main effects or interactions of lesion or NAC, although NVHLs tended to show more amphetamine activation (NVHL, 10,515 ± 2,420 cm vs. SHAM, 9,673 ± 899 cm).

One day prior to RAM, NVHL rats weighed 287.0 ± 4.5 g, compared to SHAMS (299.0 ± 12.3 g). This 4 % difference was not significant, and NAC had no effect or interactions on weight. In RAM testing, ETR showed a significant overall improvement for all rats (bins: *F*(4, 32) = 12.8, *p* < 0.001) going from 3.3 + 0.3 (bin 1) to 5.9 ± 0.4 (bin 5). However, there were no significant main effects or interactions of NVHL or NAC. Similarly, session time shortened significantly (bins: *F*(4, 32) = 31.5, *p* < 0.001), going from 278 ± 9.2 s (bin 1) to 165.8 + 17.4 s (bin 5). However, again, there were no significant NVHL or NAC-based differences or interactions on session time. Rats also consumed more FL across sessions (bins: *F*(4, 32) = 32.1, *p* < 0.001), going from 0.1 ± 0.04 (bin 1) to 5.5 ± 0.8 in (bin 5), without significant effects of lesion, NAC, or interactions.

**Fig. 6:**
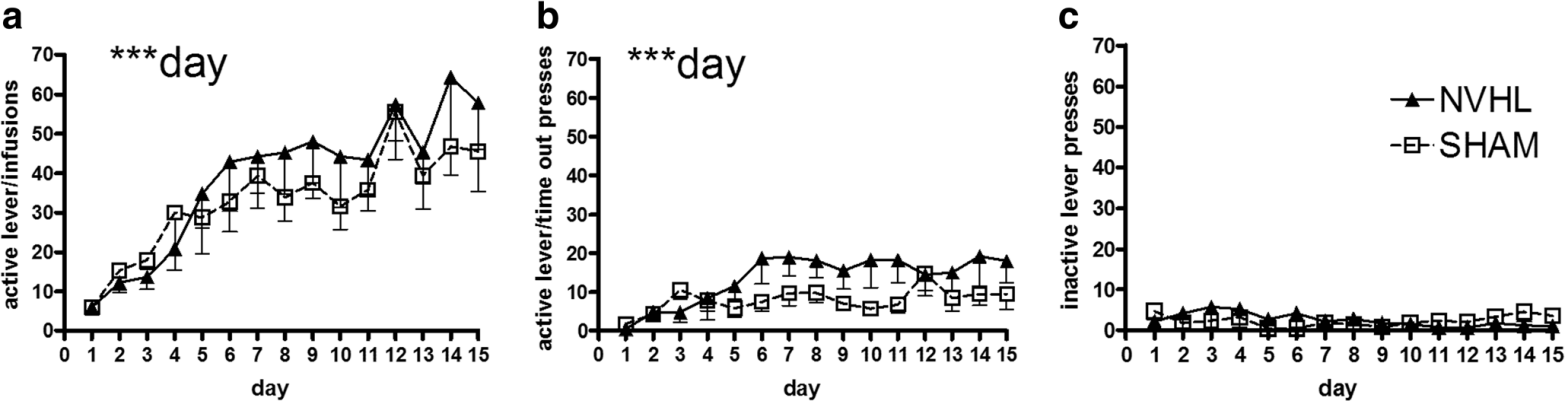
Acquisition of nicotine self-administration (experiment 2). Over 15 sessions, rats showed significant growth of bar pressing that (**a**) delivered nicotine infusions and (**b**) time-out responses on the nicotine-paired lever, with only non-significant differences based on lesion status. Responding on the non-drug paired (inactive) lever (**c**) was minimal and did not evolve over sessions. Data are depicted as means - SEMs. All significant main effects and interactions of the mixed ANOVA are shown for each dependent measure (type of lever hit) with significance levels denoted (** *p* < 0.01)

**Fig. 7:**
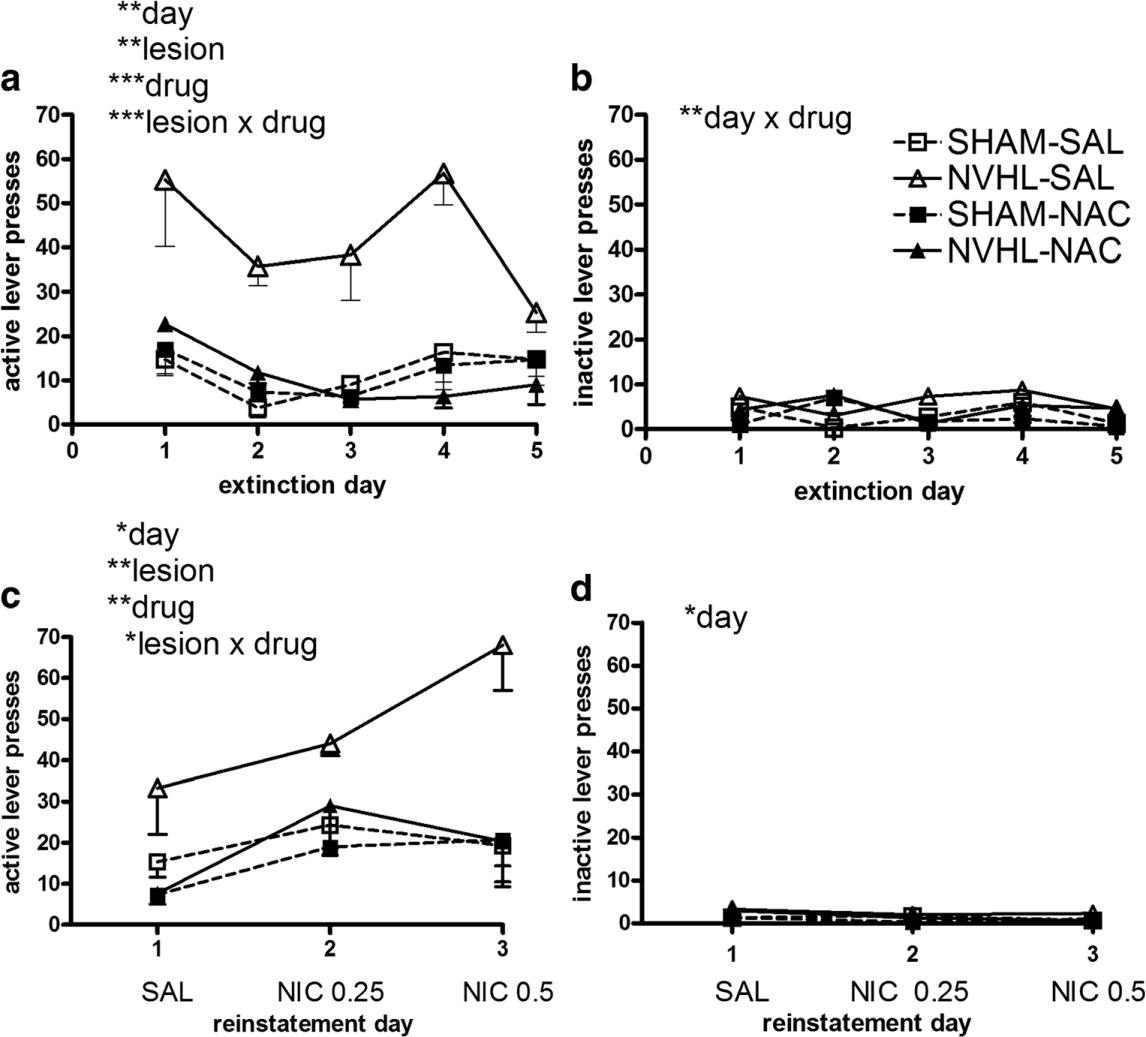
Extinction and reinstatement testing after nicotine self-administration (experiment 2). Data are plotted according to both lesion and drug treatment groups given the significant main effects and interactions of both lesion and NAC effects. In extinction on the previously nicotine-paired (active) lever (**a**) NAC specifically blunted NVHL increases in drug seeking whereas on the non-nicotine-paired (inactive) lever (**b**), there were no lesion differences and responding was low. Similarly, in reinstatement testing, on the previously nicotine-paired (active) lever (**c**), NAC also specifically blunted NVHL increases in drug seeking with no nicotine-induced responding on the non-nicotine-paired (inactive) lever (**d**). Data are depicted as means - SEMs. All significant main effects and interactions of the mixed ANOVA are shown for each dependent measure (type of lever hit) with significance levels denoted (* *p* < 0.01; **p < 0.01; ***p < 0.001)

In nicotine self-administration, rats showed good acquisition of responding for nicotine over 15 sessions (day: *F*(14,112) = 13.8, *p* < 0.001; Fig. 6a). This activity was paralleled by a smaller but still significant growth of nicotine-paired (active) lever responding that occurred during the time-out (day: *F*(14,112) = 3.2, *p* < 0.001; Fig. 6b). The inactive (non-nicotine) lever did not elicit significant growth in responding (day: *F*(14,112) = 0.8, *p* = NS; Fig. 6c). Although NVHLs tended to show greater responding on the nicotine-paired lever (Fig. 6a, b), these differences were not significant. NAC also did not impact lever responding and there were no interactions between NVHLs and NAC on any acquisition measures. In extinction, overall nicotine-paired lever pressing declined (day: *F*(4,32) = 4.8, *p* < 0.01; Fig. 7a), but now, NVHL rats showed significant resistance to extinction (lesion: *F*(1,8) = 21.7, *p* < 0.01). This was accompanied by an effect of NAC to decrease nicotine-paired lever pressing (drug: *F*(1,8) = 23.5, *p* = 0.001), that was carried by an action to specifically suppress pressing in NVHL rats to levels comparable to SHAMs (lesion × drug: *F*(1,8) = 24, *p* = 0.001). On the non-nicotine-paired lever (Fig. 7b), there was no significant overall change in responding or lesion effect, or interaction with NAC, although a day × drug interaction was detected (*F*(4,32) = 4.8, *p* < 0.01) that is likely spurious given the very low levels of all responding on the inactive lever. In reinstatement, rats showed significant increases in nicotine-paired lever responding due to nicotine pre-injections (day: *F*(2,16) = 5.5, *p* < 0.05; Fig. 7c). This effect was accompanied by overall increases in responding by NVHL rats (lesion: *F*(1,8) = 12.2, *p* < 0.01), and overall suppression of responding due to NAC (drug: *F*(1,8) = 13.2, *p* < 0.01). NAC specifically suppressed high reinstatement pressing in NVHLs (lesion × drug: *F*(1,8) = 7.70, *p* < 0.05). There were no specific interactions between day and NVHL or NAC on these measures. Non-nicotine-paired lever responding was very low (Fig. 7d) and did not differ by lesion or NAC, although some drift in responding, likely spurious in low responding, was detected (day: *F*(2,16) = 3.8, *p* < 0.05).

### RAM performance as an estimator of nicotine self-administration

**Fig. 8:**
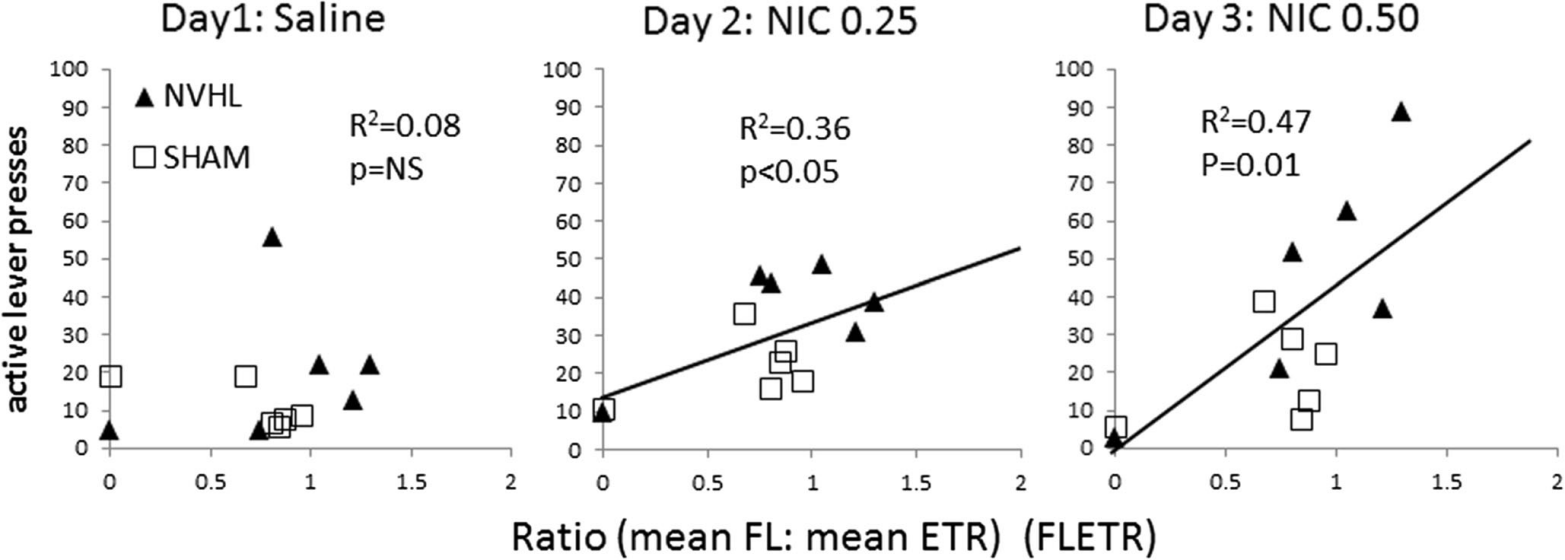
RAM performance as a linear estimator of subsequent nicotine-seeking (experiment 2). *Scatter plots* show nicotine-paired (active) lever pressing during reinstatement sessions (*y*-axis) plotted for each rat as a function of its RAM performance calculated as the ratio of mean Froot loops consumed to mean ETR recorded across the five bins (FLETR; *x*-axis). Over the population of rats in the study (*n* = 12), *R* ^2^ values and significance levels of the linear regression (*slope*) characterizing a relationship between FLETR and subsequent nicotine-seeking strengthens rightward across panels, as the relapse-provoking dose of nicotine increases. The best fit linear equations for 0.25 and 0.50-mg nicotine doses were *y* = 20.4*x* + 13.4, and *y* = 43.8*x* − 1.7, where *y* = lever pressing and *x* = FLETR

FLETR trended higher in NVHLs (0.85 ± 0.19) compared to SHAMs (0.69 ± 0.14), but not significantly. NAC also did not impact or interact with NVHLs on FLETR. As in experiment 1, mean FL and ETR were not linearly associated (*R* ^2^ = 0.23, *p* = NS). There were no significant linear relationships found between FLETR and total active lever pressing in acquisition or extinction. However, FLETR significantly estimated responding in reinstatement (Fig. 8). Although saline pre-injections did not produce lever pressing levels linearly estimated by FLETR (*R* ^2^ = 0.08, *p* = NS), pre-injections of nicotine did, in a dose-dependent way. The significant association between FLETR and levels of nicotine-induced drug seeking at the 0.25-mg/kg dose (*R* ^2^ = 0.36, *p* = 0.04) strengthened at the 0.50-mg/kg dose (*R* ^2^ = 0.47, *p* = 0.01). Outlier analyses removing any one (of *n* = 12 rats) from the latter linear regression did not result in a loss of significance. These significant linear relationships were specific to FLETR; there were no significant relationships of mean ETR or mean FL with reinstatement lever pressing.

## Discussion

In longitudinal experiments using healthy and NVHL rats, we characterized how cognitive-behavioral measures of mental illness severity (on the RAM) may predict subsequent addiction vulnerability, and how medication treatment (with NAC) initiated in adolescence may alter adult mental illness or addiction phenotypes. The two major findings are: (1) RAM measures of cognitive-behavioral profiles prior to drug exposure, are linear estimators of subsequent addiction vulnerability assessed in long-term cocaine sensitization, and nicotine self-administration; and (2) NAC has efficacy in reducing NVHL nicotine seeking after acquisition of nicotine self-administration. Although RAM learning, behavioral sensitization, and drug self-administration have long served as important paradigms for studying cognition and addiction, respectively, these experiments appear to be the first to examine them longitudinally in the same subjects and to find a quantitative-association between them. It is likely that examining both healthy and NVHL rats was important for defining this association, since NVHL rats provide a more extreme range of RAM performance (Berg et al. 2014, 2015; Chambers et al. 1996) and addiction behaviors involving cocaine and nicotine (Berg and Chambers 2008; Chambers et al. 2013; Chambers and Taylor 2004; Conroy et al. 2007).

There were two noteworthy ways that the associations between RAM and addiction phenotypes were consistent in experiments 1 and 2, despite using different drugs and behavioral paradigms. First, FLETR was a better correlate of subsequent addiction vulnerability than either FL or ETR alone. Second, the correlation of FLETR was best with greater chronicity of drug exposure, in later stages of the addicted phenotype, as elicited by drug re-exposure. This convergence suggests that FLTER is picking up on the vulnerability of PFC-striatal networks, not to the acute effects of cocaine or nicotine, but to their capacity to drive long-term neuroadaptations. Prior studies suggest that this vulnerability is related to structural and functional abnormalities within frontal-cortical-striatal networks that are post-synaptic to dopamine release and present before repeated drug exposure (Chambers et al. 2010b; Tseng et al. 2009). NVHL rats without chronic drug histories show abnormal PFC-striatal neuronal activation and gene expression patterns that are further amplified, particularly within the habit-forming striatum, after exposure to a behaviorally sensitizing regimen of cocaine (Chambers et al. 2013, 2010a). Possibly, then, FLETR serves to some extent, as an “integrated network” measure of addiction vulnerability—an estimator of PFC–hippocampal–striatal dysfunction that sets up addiction risk. It not only quantifies cognition (i.e., low ETR, reflecting impoverished PFC and hippocampal structure and function) and reward consumption (i.e., high FL consumed, related to pathological striatal function), but it computes a quotient of these variables conveying how dysfunctional one system is in proportion to the other. Hence, addiction vulnerably in mental illness and/or adolescence may reflect a mismatch involving deficient PFC cognitive and inhibitory function with subcortical-striatal hyper-reactivity to primary and drug reinforcers (Chambers et al. 2001, 2003; Mills et al. 2014). The observation from the larger experiment 1 that FL consumption is elevated in NVHL rats, while ETR (cognition) is reduced, replicates prior large group data (Berg et al. 2015) that inspired us to define and analyze FLETR as a novel RAM measure that may be relevant to understanding the linkage between mental illness and addiction. High FLETR values in NVHL rats captures two clinical attributes of schizophrenia patients: over-consumption of carbohydrate enriched-foods and cognitive deficits (McCreadie 2003). High FLETR values are also consistent with how NVHLs show increased natural-reward related impulsivity and behavioral disinhibition (Chambers et al. 2005; Chambers and Self 2002; Placek et al. 2013; Tseng et al. 2008) in parallel to PFC-based deficits in decision-making in schizophrenia patients (Heerey et al. 2008).

We also tested NAC as a treatment that hypothetically curtails abnormal glutamate signaling and/or oxidative injury that may occur in schizophrenia and/or addiction (Baker et al. 2002; Cabungcal et al. 2014; Lante et al. 2007; Brown et al. 2013; Moran et al. 2005). NAC had little impact on RAM or NVHL-phenotypes, despite prior indications that the anti-oxidant properties of NAC may normalize some behavioral and PFC-physiological abnormalities in NVHL rats (Cabungcal et al. 2014) and transgenic mice (Otte et al. 2011). However NAC did have a NVHL-specific effect in reducing nicotine addiction-related behavior, consistent with several preclinical and human studies (Asevedo et al. 2014; Carmeli et al. 2012; Grant et al. 2007; Gray et al. 2012; Knackstedt et al. 2009; Lavoie et al. 2008; Madayag et al. 2007; Moussawi et al. 2009). In particular, the way NAC suppressed nicotine-seeking after acquiring nicotine self-administration in NVHL rats, during extinction and reinstatement, are consistent with how NAC disrupts drug-induced relapse and craving in rats and humans that had already acquired addicted phenotypes (Amen et al. 2011; Ramirez-Nino et al. 2013; Zhou and Kalivas 2008). The increased seeking for nicotine observed in the extinction phase in NVHL rats is a replication (Berg et al. 2014), while the present study is the first to show increased nicotine seeking in NVHL rats in reinstatement. Further, this study is the first to show efficacy of a treatment agent for addiction in an animal model of dual diagnosis schizophrenia. NAC’s capacity to reduce expression of the addicted phenotype specifically as it is accentuated in the NVHL model, supports a common biological basis for addiction and mental illness at the level of glutamatergic neurotransmission.

Further studies are needed to address many new questions that arise from these data such as whether adolescent dosing of NAC prior to, or during addictive drug exposure optimizes efficacy, or, whether FLETR on the RAM can also estimate addiction risk in cocaine self-administration. The use of NAC at a single dose generates promising lead information for more adequate pharmacological characterizations of glutamatergic mechanisms that may link mental illness and addiction. Our findings provide a parallel to clinical cognitive testing of PFC function (e.g., Wisconsin Card Sort, Iowa Gambling task, Delay Discounting) used to quantify severity of both mental illness and addiction vulnerability (Bechara et al. 1994; Bickel and Johnson 2003; Weinberger et al. 1985). By suggesting that a quantifiable estimator of addiction risk may emerge from a quotient of two variables that measure primitive reward motivation (striatal circuits) and cognition (PFC–hippocampal networks; Bahner et al. 2015), the present results suggest the utility of analogous compound variables in humans to determine dual diagnosis vulnerability and selectively implement pharmacological preventions. When paired with functional neuroimaging approaches (Drobac and Hulvershorn 2014; Hulvershorn et al. 2013), such testing in adolescents and young adults could inform clinical decision-making on novel treatments that reduce addictions in the most vulnerable patients.
